# Internal and external cooling methods and their effect on body temperature, thermal perception and dexterity

**DOI:** 10.1371/journal.pone.0191416

**Published:** 2018-01-22

**Authors:** Matthew J. Maley, Geoffrey M. Minett, Aaron J. E. Bach, Stephanie A. Zietek, Kelly L. Stewart, Ian B. Stewart

**Affiliations:** Institute of Health and Biomedical Innovation, School of Exercise and Nutrition Sciences, Queensland University of Technology, Brisbane, Australia; St. Joseph’s Hospital and Medical Center, UNITED STATES

## Abstract

**Objective:**

The present study aimed to compare a range of cooling methods possibly utilised by occupational workers, focusing on their effect on body temperature, perception and manual dexterity.

**Methods:**

Ten male participants completed eight trials involving 30 min of seated rest followed by 30 min of cooling or control of no cooling (CON) (34°C, 58% relative humidity). The cooling methods utilised were: ice cooling vest (CV_0_), phase change cooling vest melting at 14°C (CV_14_), evaporative cooling vest (CV_EV_), arm immersion in 10°C water (AI), portable water-perfused suit (WPS), heliox inhalation (HE) and ice slushy ingestion (SL). Immediately before and after cooling, participants were assessed for fine (Purdue pegboard task) and gross (grip and pinch strength) manual dexterity. Rectal and skin temperature, as well as thermal sensation and comfort, were monitored throughout.

**Results:**

Compared with CON, SL was the only method to reduce rectal temperature (P = 0.012). All externally applied cooling methods reduced skin temperature (P<0.05), though CV_0_ resulted in the lowest skin temperature versus other cooling methods. Participants felt cooler with CV_0_, CV_14_, WPS, AI and SL (P<0.05). AI significantly impaired Purdue pegboard performance (P = 0.001), but did not affect grip or pinch strength (P>0.05).

**Conclusion:**

The present study observed that ice ingestion or ice applied to the skin produced the greatest effect on rectal and skin temperature, respectively. AI should not be utilised if workers require subsequent fine manual dexterity. These results will help inform future studies investigating appropriate pre-cooling methods for the occupational worker.

## Introduction

There is a fine balance between maintaining productivity and safety of individuals working in environmental extremes. While high environmental temperatures will result in body heat gain at rest [1], the additional heat production from physical activity further hastens heat storage in these environments [2]. The resultant increase in body temperature is associated with reductions in work capacity [3–5]. Further, continuation of physical activity in these environments may result in serious heat-related injury or even death [6].

Alleviating thermal strain during work in hot conditions may be possible with some form of cooling before work. Broadly, an individual may utilise external or internal cooling methods [7,8]. External cooling involves the application of a cooling medium (e.g. cold water, ice vest) to an individual’s skin. The cooled skin may subsequently cool the cutaneous circulating blood and abate the rise in deep body temperature during work [9,10]. Internal cooling involves an individual ingesting (e.g. ice slushy) or inhaling (e.g. cold air) a medium capable of cooling. Both internal cooling methods may result in a decrease in deep body temperature, with little change in skin temperature [7].

Using internal or external cooling strategies before work (i.e. pre-cooling) may improve performance or reduce thermal strain during fixed exercise intensity [11–16]. Specific to occupational workers, Tokizawa [17] was able to reduce participants’ thermal strain during walking in the heat (37°C, 40% relative humidity) dressed in a chemical protective garment with the use of 30 min of pre-cooling using a fan and water sprayed over the entire body. Similarly, a reduction in thermal strain during work was achieved with ice slushy ingestion before walking in the heat (39°C, 18% relative humidity) dressed in wildland firefighting garments [18].

Deciding on the cooling method to utilise involves consideration of the effectiveness (of cooling), access, transport, time and cost [14–16]. Within occupational settings, particularly emergency response teams, the unknown location, resources and time available are all factors that may influence the choice of pre-cooling method. At present, there are no clear guides and limited data in an occupational context on the optimal length of time a cooling method should be applied for before work. This may result in individuals undertaking a cooling protocol for a longer period than necessary. Additionally, individuals should also consider any detrimental impact the type of pre-cooling method may have on subsequent occupational task requirements [19]. For example, while arm immersion may be successful in reducing thermal strain [20], cooling the arms may subsequently negatively impact manual dexterity [21].

Unfortunately, many investigations assessing the effect of cooling on body temperature are limited by the number of pre-cooling methods compared. Investigations often compare a single pre-cooling method against a control of no cooling [22,23], where others have compared only two [12,24,25] or three [15,26] pre-cooling methods. Therefore, the aim of the present study is to compare a broad range of pre-cooling methods as a repeated measures design, focusing on their effect on body temperature and manual dexterity. As the present study focuses on those who may utilise cooling before work in an occupational setting, the opportunity to assess a potentially novel internal cooling method, Heliox inhalation, is investigated. It is hypothesised that external cooling methods will lower skin temperature, while ice slushy ingestion will lower deep body temperature. Additionally, it is hypothesised manual dexterity will be negatively impacted by arm immersion only.

## Methods

This study was approved by the Queensland University of Technology’s Human Research Ethics Committee and complied with standards set in the Declaration of Helsinki. The participants were made aware of the purpose, procedures and risks of the study before giving their informed written consent. Ten male participants volunteered; their physical characteristics were as follows (mean [SD]): 23 (3) years of age, height of 180 (6) cm, body mass of 86 (7) kg and body fat of 26 (8) %. All participants were non-smokers and free from any vascular, blood and respiratory conditions. Participants were instructed to refrain from alcohol, caffeine and strenuous exercise in the 24 h preceding each visit to the laboratory.

### Experimental sessions

Following familiarisation, participants attended the laboratory for eight experimental sessions at the same time of day, separated by a minimum of 24 h. Within each visit participants remained seated in a climate controlled chamber (dry bulb temperature 34.4 [0.5] °C, wet bulb temperature 27.6 [0.8] °C, relative humidity 58 [4] %) for a baseline period of 30 min followed by 30 min of cooling application or control (CON) of no cooling (detailed below). Environmental temperature and humidity were recorded throughout and measured using a wet bulb globe thermometer (QUESTemp 36, 3M, Minnesota, USA). The order of testing was randomised using a random number generator (Research Randomiser, v4, Social Psychology Network). Participants wore the same t-shirt, shorts and shoes for each trial. Where applicable, the cooling garment was applied over participant’s clothing.

Immediately before and after cooling, participants were asked to perform a battery of tests to assess their manual dexterity. These tests included: 1) Purdue Pegboard test, 2) grip strength and 3) pinch strength. The Purdue Pegboard test (Model 32020, Lafayette Instrument, Lafayette, USA) was utilised to assess fine manual dexterity where participants are required to place pins into the right- or left-hand row of vertical holes in the board for 30 s. Separated by 30 s, participants started with their dominant hand first, followed by their non-dominant hand and then each hand simultaneously. All three scores were summed. Following this, grip and then pinch strength was assessed using a dynamometer (Digital Multi-Myometer, MIE Medical Research Ltd., Leeds, UK). For grip strength, participants were measured with the shoulder at 0° flexion, elbow at 90° and their wrists in a neutral position. For pinch strength, the shoulder and elbow were positioned the same as for grip strength, whereas the wrist was pronated with participants pinching the dynamometer between the thumb and index finger only. Peak force (N) of grip and pinch strength were averaged over three attempts that were held for 3 s, each separated by 60 s.

### Cooling methods

#### Cooling vests

Three different cooling vests were tested: 1) an ice-based cooling vest (CV_0_), stored in a -20°C freezer (ICEEPAK Australia, Mooloolaba, Australia); 2) a non-ice-based cooling vest with a melting temperature of 14°C (CV_14_), stored in a 4°C fridge (KewlFit, Model 6626-PEV, TechNiche, Vista, USA); 3) an evaporative cooling vest, immersed in water (~17°C) for at least 2 min immediately before the commencement of cooling (CV_EV_) (KewlShirt, Model 6201, TechNiche, Vista, USA).

#### Arm immersion (AI)

Participants sat in a collapsible chair (Kore Kooler Rehab Chair, DQE, Indianapolis, USA) with disposable bags placed in the troughs built into the armrests and filled with cold water (10.4 [0.7] °C) immediately before arm immersion. Participants were instructed to fully immerse their hands and forearms to approximately 5 cm above the medial epicondyle of the humerus. No attempt was made to maintain the water temperature which rose to 19.9 (0.8) °C by the end of cooling.

#### Water-perfused suit (WPS)

Participants donned a three-piece portable battery-operated water-perfused suit (WPS; BCS4 Cooling System, Med-Eng, Ottawa, Canada) that covered the entire body, except the face, hands and feet. The WPS consists of tubing sewn into a stretchable pullover, trousers and hood. Water was circulated at ~375 mL·min^–1^ from an integrated portable pump (Delta Wing Pump, Med-Eng, Ottawa, Canada) connected to a specifically designed bottle which initially contained 90% ice and 10% water. This resulted in ~10°C water entering the WPS when first turned on. Like the AI protocol, no attempt was made to maintain water temperature.

#### Heliox (HE)

Participants breathed a mixture of 21% O_2_ and 79% He (BOC Limited, Ipswich, Australia) from a Douglas bag attached to a two-way T-shape non-rebreathing valve (Model 2700, Hans-Rudolph, Shawnee, USA) and head support (Model 2726, Hans-Rudolph, Shawnee, USA). The temperature of the heliox mixture was matched to the ambient temperature (i.e. 35°C).

#### Ice slushy (SL)

Participants ingested 7.5 g·kg^–1^ of ice slushy (-2.2 [0.4] °C) at a rate of 1.25 g·kg^–1^ every 5 min to standardise the ingestion rate [11]. Each drink was prepared using a slushy machine (Model SSM-180, ICETRO, Incheon, South Korea) with the same flavouring used for each participant (Fruchilla Natural 99% Fruit Juice, The Slushie Specialists, Bentleigh East, Australia).

### Measurements and calculations

Body composition was measured using dual-energy X-ray absorptiometry (Lunar Prodigy, GE Healthcare Lunar, Madison, USA) and analysed using dedicated software (enCORE, version 9, GE Healthcare Lunar, Madison, USA). Pre-trial hydration status was confirmed by urine specific gravity (PAL 10s, ATAGO, Tokyo, Japan) of <1.020 [27]. If participants provided a sample >1.020 they were given an additional 500 mL of tap water, which was consumed 30 min before the commencement of the trial.

The experiments followed the termination criteria set in ISO 12894 [28]; however, no participants terminated early. Deep body temperature was estimated from rectal temperature (T_rec_) using a thermistor (YSI 400, DeRoyal, Knox, USA) self-inserted 12 cm beyond the anal sphincter and recorded using a data logger (Squirrel 2020 series, Grant Instruments, Cambridge, UK). Mean skin temperature (${\bar{T}}_{msk}$) was estimated using wireless iButton thermocrons (DS1922L-F50 iButtons, Maxim Integrated, San Jose, USA) attached to eight sites using a single piece of adhesive tape (Premium Sports Tape, AllCare, Kumeu, New Zealand) and calculated as (ISO 9886 [29]):

$$\bar{T}{}_{msk}\;=\;0.07T_{head}+0.175T_{scapula}+0.175T{}_{chest}+0.07T_{upperarm}+0.07T_{forearm}+0.05T_{hand}+0.19T_{thigh}+0.20T_{calf}$$

Mean body temperature (${\bar{T}}_{b}$) was calculated as [30]:

$${\bar{T}}_{b}\;=\;0.8T_{rec}+0.2{\bar{T}}_{msk}$$

Both T_rec_ and ${\bar{T}}_{msk}$ were recorded at 2 s intervals, averaged per min and analysed every 5 min. The temperature of SL and water during AI were measured using a calibrated thermometer (TL-1W, ThermoProbe, Pearl, USA). Thermal sensation was assessed using a modified scale [31], where 1 had the anchor of ‘unbearably cold’, 7 ‘neutral’ and 13 ‘unbearably hot’. Similarly, thermal comfort was assessed using a modified scale [31], where 1 had the anchor of ‘comfortable’ and 5 ‘extremely uncomfortable’. Both thermal sensation and thermal comfort were recorded every 5 min.

### Statistical analyses

Statistical analyses were conducted using SPSS version 23 for Windows (IBM Corporation, New York, USA). An α of 0.05 was used to determine statistical significance. Data were assessed for normality with a Shapiro-Wilk test and visual inspection of data (e.g. boxplots). Baseline values between cooling methods were compared using a one-way repeated measures analysis of variance. As baseline physiological responses were similar between cooling methods (see Results), delta (Δ) T_rec_, ${\Delta \bar{T}}_{msk},\;{\Delta \bar{T}}_{b}$ and thermal sensation and comfort were compared between cooling methods and CON (i.e. trial) across time using a two-way repeated measures analysis of variance.

Additionally, ΔT_rec_, ${\Delta \bar{T}}_{msk}$ and ${\Delta \bar{T}}_{b}$ were analysed within trial over time, with time points analysed sequentially. That is, the first comparison was always made between baseline and subsequent time points (e.g. 0 vs 5, 0 vs 10). When a significant difference was observed (e.g. 10^th^ min), that time point was compared to the next time point (e.g. 10 vs 15). These steps were repeated until all pairwise comparisons were conducted (i.e. six for each trial). Hand T_sk_ and manual dexterity variables between cooling methods and CON were analysed before and after cooling using a two-way repeated measures analysis of variance. When a main effect or significant interaction was achieved, paired samples *t*-tests were conducted with Bonferroni adjustments applied for multiple comparisons.

Effect sizes were calculated for pairwise comparisons using Cohen’s *d*_*av*_ [32] and interpreted as small (0.2–0.4), moderate (0.5–0.7) or large (≥0.8) [33,34]. All data in text, figures and tables are presented as mean and SD.

## Results

### Physiological responses

All baseline physiological variables were similar between trials (Table 1; P > 0.05).

**Table 1:** Baseline rectal, mean skin and mean body temperature.

	T_rec_ (°C)	${\bar{T}}_{msk}$(°C)	${\bar{T}}_{b}$(°C)
**CON**	37.2 (0.1)	34.8 (0.4)	36.7 (0.1)
**CV_0_**	37.3 (0.2)	34.7 (0.6)	36.8 (0.3)
**CV_14_**	37.4 (0.2)	35.0 (0.4)	36.9 (0.2)
**CV_EV_**	37.3 (0.2)	34.8 (0.3)	36.8 (0.1)
**AI**	37.3 (0.2)	34.9 (0.6)	36.8 (0.2)
**WPS**	37.3 (0.2)	34.7 (0.4)	36.7 (0.2)
**SL**	37.3 (0.2)	34.8 (0.6)	36.8 (0.3)
**HE**	37.3 (0.2)	35.1 (0.5)	36.8 (0.2)

#### Delta rectal temperature

**Fig 1:**
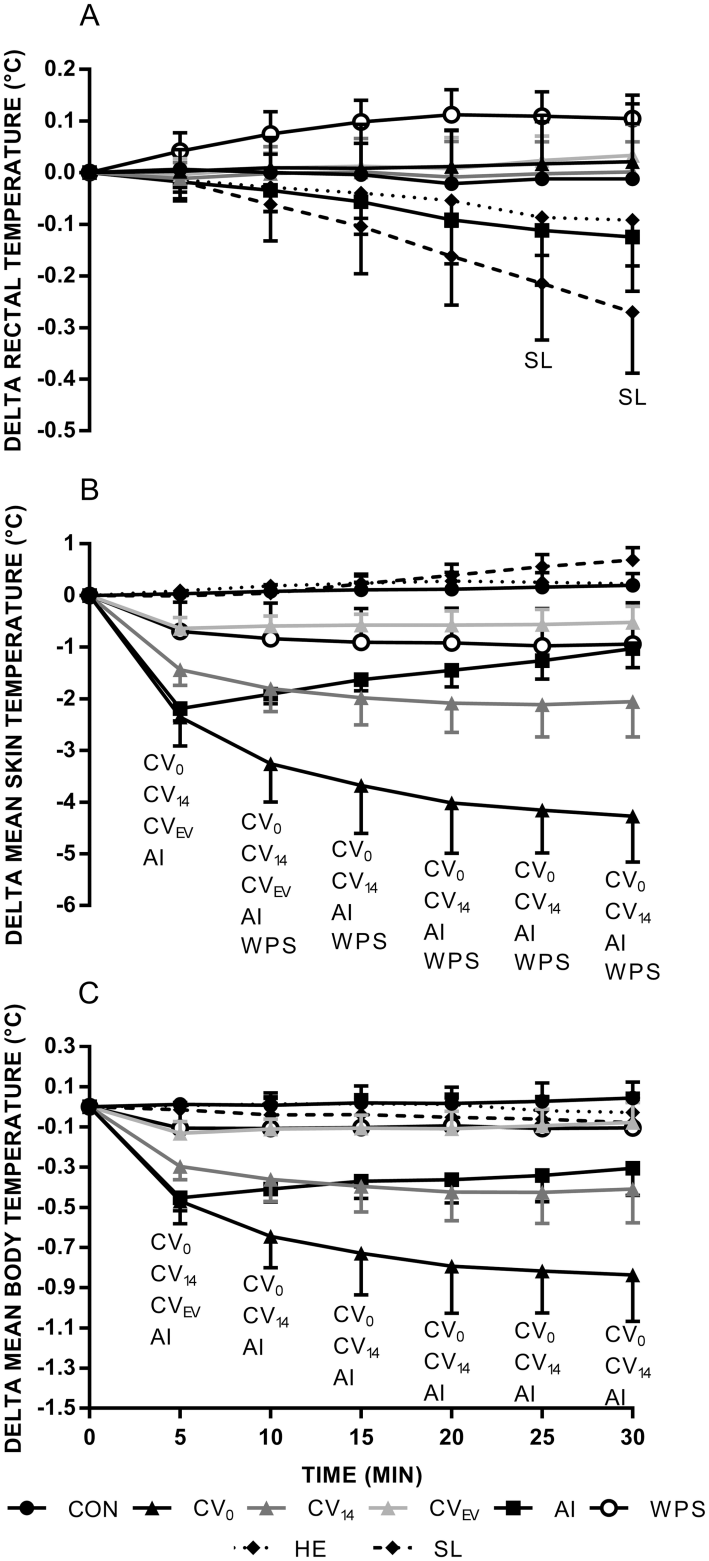
Delta rectal (A), mean skin (B) and mean body temperature (C) throughout cooling. Note: Abbreviations denote significant (P < 0.05) difference from CON.

There was a significant main effect for trial (P < 0.001), time (P = 0.001) and interaction (P < 0.001). Pairwise comparisons of the main effect for trial revealed ΔT_rec_ for WPS was significantly different versus CV_EV_, AI, HE and SL. Within trial analyses revealed T_rec_ was lowered throughout cooling in SL (0 > 20 > 25 > 30 min), while it increased in WPS (0 < 10 < 15 min). Pairwise comparisons of the interaction revealed CON ΔT_rec_ significantly differed from SL at minute 25 (Fig 1A; P = 0.048, *d*_*av*_ = 1.8) and 30 (P = 0.012, *d*_*av*_ = 2.3).

#### Delta mean skin temperature

There was a significant main effect for trial (P < 0.001), time (P = 0.036) and interaction (P < 0.001). Pairwise comparisons of the main effect for trial revealed Δ${\bar{T}}_{msk}$ differed between cooling methods (CV_0_ < CV_14_, AI < CV_EV_ < CON, HE, SL). WPS produced a lower ${\bar{T}}_{msk}$ compared with HE and SL but was greater than CV_0_. Within trial analyses revealed $\Delta {\bar{T}}_{msk}$ increased over time for CON (0 < 30 min), SL (0 < 20 < 25 < 30 min) and HE (0 < 15 min), and lowered over time for CV_0_ (0 > 5 > 10 > 20 min), CV_14_ (0 > 5 > 10 > 20 min) and CV_EV_ (0 > 5 min). Initially AI lowered ${\bar{T}}_{msk}$ but subsequently rose (0 > 5 < 10 < 15 < 25 < 30 min). Pairwise comparisons of the interaction revealed CON $\Delta {\bar{T}}_{msk}$ differed from CV_0_ (Fig 1B; P < 0.001, d_av_ = 7.1–9.1), CV_14_ (P < 0.001, *d*_*av*_ = 5.8–8.1), CV_EV_ (P < 0.001, *d*_*av*_ = 3.4–5.0), AI (P < 0.001, *d*_*av*_ = 5.3–14.7) and WPS (P = 0.032–0.039, *d*_*av*_ = 2.5–2.7) throughout cooling.

#### Delta mean body temperature

There was a significant main effect for trial (P < 0.001), time (P = 0.010) and interaction (P < 0.001). Pairwise comparisons of the main effect for trial revealed $\Delta {\bar{T}}_{b}$ was significantly lower in CV_0_ than all other trials (P < 0.05) except CV_14_ (P > 0.05). ${\bar{T}}_{b}$ was lower in CV_14_ and AI compared with CV_EV_, HE, SL and CON (P < 0.004). WPS did not differ from CON (P > 0.05). Within trial analyses revealed $\Delta {\bar{T}}_{b}$ was lowered over time for CV_0_ (0 > 5 > 10 > 25 min), CV_14_ (0 > 5 min) and CV_EV_ (0 > 5 min). AI initially lowered ${\bar{T}}_{b}$ but was soon followed by a subsequent increase (0 > 5 < 10 min). Pairwise comparisons of the interaction revealed CON $\Delta {\bar{T}}_{b}$ differed from CV_0_ (Fig 1C; P < 0.001, *d*_*av*_ = 5.1–6.8), CV_14_ (P < 0.001, *d*_*av*_ = 3.7–6.5), CV_EV_ (P = 0.001, *d*_*av*_ = 3.3) and AI (P = 0.001–0.002, *d*_*av*_ = 3.2–9.9) throughout cooling.

#### Hand skin temperature

There was a significant main effect for trial (P < 0.001), time (P = 0.010) and interaction (P < 0.001). Hand T_sk_ was similar between cooling methods before cooling (CON: 35.0 [0.5] °C). Pairwise comparisons revealed at the end of cooling hand T_sk_ was cooler following AI (19.6 [1.0] °C) compared with CON (35.3 [0.3] °C; P < 0.001, *d*_*av*_ = 24.1).

### Manual dexterity

#### Purdue pegboard

There was a significant main effect for trial (P = 0.031), time (P = 0.004) and interaction (P < 0.001). Pairwise comparisons revealed participants achieved a lower score following AI compared with CON (Table 2; P = 0.001, *d*_*av*_ = 1.7).

**Table 2:** Purdue pegboard, grip strength and pinch strength before and after cooling.

	Purdue (score)		Grip Strength (N)		Pinch Strength (N)	
	Before	After	Before	After	Before	After
**CON**	67 (5)	64 (8)	522 (61)	511 (55)	81 (17)	70 (16)
**CV_0_**	62 (4)	65 (3)	517 (61)	478 (45)	76 (7)	74 (11)
**CV_14_**	64 (11)	66 (9)	498 (52)	477 (46)	78 (13)	74 (13)
**CV_EV_**	62 (6)	62 (5)	493 (49)	476 (58)	78 (14)	74 (12)
**AI**	66 (4)	49 (5) ^a^	500 (57)	485 (58)	84 (17)	82 (19)
**WPS**	67 (5)	66 (5)	525 (66)	509 (48)	74 (17)	70 (18)
**SL**	66 (5)	64 (4)	521 (66)	500 (52)	73 (15)	71 (12)
**HE**	65 (6)	66 (5)	518 (51)	525 (51)	78 (17)	74 (19)

^a^Significant difference from CON after cooling (P < 0.05).

N, Newtons.

#### Grip and pinch strength

For grip strength, there was a significant main effect for trial (P = 0.048) and time (P = 0.004), but no interaction (P = 0.272). Pairwise comparisons revealed participants achieved greater scores before versus after cooling (Table 2; P = 0.028, *d*_*av*_ = 0.4).

For pinch strength, there was a significant main effect for trial (P = 0.034) and time (P = 0.001), but no interaction (P = 0.063). Like grip strength, pairwise comparisons revealed participants achieved greater scores before versus after cooling (Table 2; P = 0.001, *d*_*av*_ = 0.3).

### Perceptual responses

**Fig 2:**
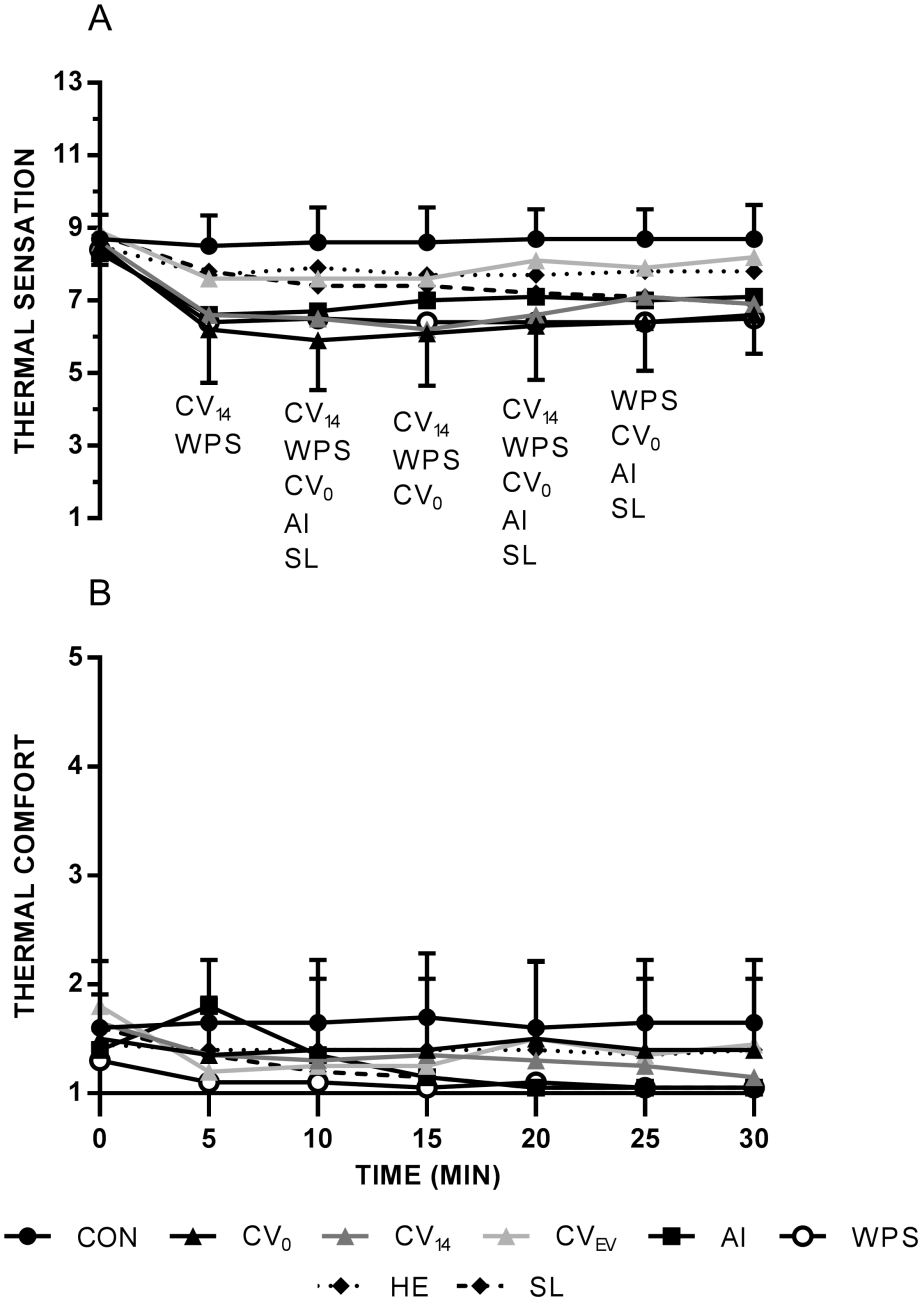
Thermal sensation (A) and comfort (B) throughout cooling. Note: For reader clarity, SD is shown for CON and CV_0_ only. Abbreviations denote significant (P < 0.05) difference from CON.

For thermal sensation, there was a significant main effect for trial (P < 0.001), time (P = 0.010) and interaction (P < 0.001). Pairwise comparisons of the main effect for trial revealed compared with CON thermal sensation was lower in CV_0_, CV_14_, AI, SL and WPS (P < 0.05). Further, thermal sensation was lower during the WPS trial compared with CV_EV_ and HE (P < 0.05). Pairwise comparisons for the interaction revealed CON differed from CV_0_ (Fig 2A; P = 0.027–0.047, *d*_*av*_ = 2.1–2.3), CV_14_ (P = 0.09–0.018, *d*_*av*_ = 2.3–2.5), AI (P = 0.020–0.033, *d*_*av*_ = 2.0–2.8), SL (P = 0.017–0.047, *d*_*av*_ = 1.6–2.3) and WPS (P = 0.007–0.045, *d*_*av*_ = 2.3–2.5).

For thermal comfort, there was a significant main effect for trial (P = 0.001), time (P = 0.001) and interaction (P < 0.001). However, no significant pairwise comparisons were revealed for the main effect of cooling method or interaction (Fig 2B; P > 0.05).

## Discussion

This study adds to the current literature by providing data from seven methods that could be utilised for cooling before work and their effect on body temperature, dexterity and thermal perception. The primary findings were that SL was the only cooling method to reduce deep body temperature (Fig 1A) and all external cooling methods reduced ${\bar{T}}_{msk}$ (Fig 1B). AI reduced Purdue pegboard performance by 23%, with no effect on grip or pinch strength (Table 2).

Normothermic individuals who have ingested SL have experienced a decrease in T_rec_ between 0.3°C to 0.66°C [11,24,25,35,36]. The magnitude of T_rec_ reduction is similar to previous studies when an ice slushy was consumed in a warm environment (>30°C) [24,25,36]. In contrast, Siegel et al. [11] asked participants to consume an ice slushy in an ambient temperature of 24°C and reported a 0.66°C reduction in T_rec_. It is likely that when an ice slushy is consumed in a warm environment there appears to be a more modest reduction in T_rec_ due to a reduced dry heat loss.

Occupational settings, such as firefighting, may sometimes require breathing apparatus filled with compressed air, which could be replaced with other gas mixtures. The effect of inhaled HE on deep body temperature in animal models is equivocal [37–39]. Data from human studies are limited, with one study reporting a reduction in deep body temperature when inhaling heliox within a hyperbaric environment [40]. However, HE did not reduce deep body temperature in the present study (Fig 1A). The reason for this may relate to the gas characteristics. Thermal conductivity of helium is six times that of nitrogen [41], however, respiratory heat loss is dependent on thermal capacity [42,43]. Thus, despite a greater specific heat capacity for helium versus nitrogen, helium has a lower density [41] and therefore a lower thermal capacity (i.e. thermal capacity = specific heat × density).

As hypothesised, all external cooling methods reduced ${\bar{T}}_{msk}$ but did not reduce T_rec_. Previous research has demonstrated that application of an ice vest to normothermic individuals does not reduce deep body temperature [9,12,26]. Similarly, non-ice based cooling vests consistently shows no reduction in deep body temperature [12,44,45], aligning with the present study’s findings.

Considering work capability (performance or capacity) is limited, in part, by high deep body temperatures [46], the pre-cooling method that has the greatest effect on deep body temperature would appear the most optimal choice. Despite no T_rec_ reduction within 30 min of external cooling in the present study, these methods may abate the rise in body temperature during subsequent work. This is supported by research that has shown external cooling to reduce thermal strain when applied before work in the heat in protective clothing [17]. Thus, considering SL and CV_0_ provided the greatest cooling (physiologically and perceptually) and are relatively accessible, future work should focus on utilising these cooling methods before work in the heat and protective clothing.

Pre-cooling protocols are varied in their length of application, ranging from 20 min [26,47] to 45 min [12] with little rationale. The present study demonstrated CV_0_ and CV_14_ resulted in ${\bar{T}}_{msk}$ changes within 5 min and further reductions were observed up until the 20^th^ min. However, CV_EV_ only produced a reduction in ${\bar{T}}_{msk}$ within 5 min, with AI initially lowering ${\bar{T}}_{msk}$ and then subsequently increasing. Practically, if an individual has only 5 min for pre-cooling then these methods will result in lower ${\bar{T}}_{msk}$, but CV_EV_ and AI will not result in further benefits past this. As no further reductions in ${\bar{T}}_{msk}$ were observed following 20 min of cooling using the ice or non-ice cooling vest it is suggested this is an optimal cooling duration.

Colder hand and finger T_sk_ are associated with reductions in manual dexterity [48]. Previous research has demonstrated extremity cooling results in a reduction in both fine (e.g. Purdue pegboard) [49,50] and gross task performance (e.g. grip strength) [51,52]. In the present study, however, AI lowered hand T_sk_ and adversely affected Purdue pegboard performance, but did not affect grip or pinch strength (Table 2). The reason for the lack of effect of cold extremities on grip and pinch strength in the present study is unclear, however, it is obvious that fine tasks are most affected by cold extremities.

While the present study reports the responses to a broad range of pre-cooling methods, the results may not accurately translate to all individuals. Factors such as age, sex, body mass, surface area-to-mass ratio and body composition may influence responses to cooling [53]. Despite this, while the magnitude of responses may differ between distinctive groups, the pre-cooling methods that are most effective may not.

It is concluded that while SL will lower deep body temperature, the external cooling methods used in this study will lower ${\bar{T}}_{msk}$, with no reductions in deep body temperature. The user should be mindful that using external cooling methods similar to the present study provides no further ${\bar{T}}_{msk}$ cooling past 20 min. Finally, cooling the extremities may compromise manual dexterity, and therefore individuals requiring fine manual dexterity should opt for an alternative pre-cooling method.
